# Hyperactive sensorimotor cortex during voice perception in spasmodic dysphonia

**DOI:** 10.1038/s41598-020-73450-0

**Published:** 2020-10-14

**Authors:** Yuji Kanazawa, Yo Kishimoto, Ichiro Tateya, Toru Ishii, Tetsuji Sanuki, Shinya Hiroshiba, Toshihiko Aso, Koichi Omori, Kimihiro Nakamura

**Affiliations:** 1 Department of Otolaryngology-Head and Neck Surgery, Graduate School of Medicine, Kyoto University, Kyoto, Japan; 2 Department of Otolaryngology, Shiga Medical Center for Children, Moriyama, Japan; 3 Department of Otolaryngology, Shiga Medical Center Research Institute, Moriyama, Japan; 4 Department of Otolaryngology, Fujita Health University School of Medicine, Nagoya, 470-1192 Japan; 5 Human Brain Research Center, Graduate School of Medicine, Kyoto University, Kyoto, Japan; 6 Department of Otolaryngology-Head and Neck Surgery, Kumamoto University, Kumamoto, Japan; 7 Department of Otolaryngology-Head and Neck Surgery, Nagoya City University Graduate School of Medical Sciences, Nagoya, Japan; 8 HIROSHIBA ENT Clinic/Isshiki Memorial Voice Center, Kyoto, Japan; 9 Section of Systems Neuroscience, National Rehabilitation Center Research Institute, Tokorozawa, Japan

**Keywords:** Neuroscience, Biomarkers, Medical research, Neurology, Nanoscience and technology

## Abstract

Spasmodic dysphonia (SD) is characterized by an involuntary laryngeal muscle spasm during vocalization. Previous studies measured brain activation during voice production and suggested that SD arises from abnormal sensorimotor integration involving the sensorimotor cortex. However, it remains unclear whether this abnormal sensorimotor activation merely reflects neural activation produced by abnormal vocalization. To identify the specific neural correlates of SD, we used a sound discrimination task without overt vocalization to compare neural activation between 11 patients with SD and healthy participants. Participants underwent functional MRI during a two-alternative judgment task for auditory stimuli, which could be modal or falsetto voice. Since vocalization in falsetto is intact in SD, we predicted that neural activation during speech perception would differ between the two groups only for modal voice and not for falsetto voice. Group-by-stimulus interaction was observed in the left sensorimotor cortex and thalamus, suggesting that voice perception activates different neural systems between the two groups. Moreover, the sensorimotor signals positively correlated with disease severity of SD, and classified the two groups with 73% accuracy in linear discriminant analysis. Thus, the sensorimotor cortex and thalamus play a central role in SD pathophysiology and sensorimotor signals can be a new biomarker for SD diagnosis.

## Introduction

Spasmodic dysphonia (SD) is a type of idiopathic focal dystonia characterized by an involuntary laryngeal muscle spasm during voice production^1,2^. Unlike other types of dystonic syndromes, such as cervical dystonia and writer’s cramp, SD is poorly recognized in the medical community and often misdiagnosed as a psychogenic condition^3,4^ or as other neurological voice disorders^5,6^. In fact, accurate diagnosis of SD is elusive and challenging for most clinicians, typically requiring careful and coordinated multi-disciplinary investigation, including a clinical history questionnaire, speech assessment, laryngoscopy, and neuroimaging^7^. For example, patients may need to see four different physicians over more than four years to receive a final diagnosis of SD, probably because no systematic protocol for clinical assessment is established for the disease^8^. It is therefore of vital significance to develop objective diagnostic criteria and biomarkers for initiating appropriate therapeutic interventions in early stage of the disease. Like other types of dystonia, muscular hyperactivity in SD has been associated with abnormal sensory-motor integration^1,9^, yet its precise neuroanatomical locus remains elusive.

A few previous neuroimaging studies have suggested that SD is associated with abnormal sensorimotor integration in the primary sensorimotor cortex, basal ganglia, thalamus, and cerebellum^9–11^. These findings seem to be partially consistent with some recent studies using transcranial magnetic stimulation (TMS), which showed changes in motor cortical excitability in SD patients^12,13^. However, it remains unclear to what extent the observed activations in those cortical and subcortical structures reflect the endogenous pathophysiological mechanisms of the disease, since most of the activation differences between SD patients and healthy controls were obtained by measuring brain activity during *voice production*. That is, such activation differences can be attributed to the differences in the nature of vocalization between SD patients and controls during voice production, obviously because SD patients, unlike healthy controls, should have much difficulty in vocalization, i.e., the act of voice production in itself should be much more limited and effortful for SD patients than for controls, which engages the motor, auditory and cognitive systems differently in the two groups and thereby creates a large confounding factor in between-group comparison analyses.

This may explain the fact that the reported patterns of neural activation are rather inconsistent across those previous studies. For example, a positron emission tomography study by Ali et al.^10^ observed increased activation in the ventral sensorimotor, auditory and anterior cingulate cortices, insula, and cerebellum and decreased activation in the supplementary motor area (SMA). Using functional magnetic resonance (fMRI), however, Haslinger et al.^11^ observed reduced activation in the primary sensorimotor, premotor, and sensory association cortices. Interestingly, moreover, Simonyan and Ludlow^9^ deliberately trained healthy participants to “imitate” the typical voice patterns of SD patients and measured their brain activity using fMRI. The authors observed increased activation in the primary sensorimotor cortex, insula, superior temporal gyrus, basal ganglia, thalamus and cerebellum in SD patients as compared to controls. While this experimental manipulation largely allowed matching of the amount of vocal outputs between SD patients and controls, the observed neural effects may reflect a strategic recourse to other neurocognitive resources, because healthy volunteers would exert highly unnatural and effortful control over the normal speech production system.

More recently, functional connectivity analysis using resting-state fMRI has been used as an alternative imaging method to overcome the inherent problems in comparing SD patients with healthy or non-SD participants. In particular, Battistella et al.^14^ showed abnormal functional connectivity within the sensorimotor and frontoparietal networks in patients compared with healthy individuals. While resting-state fMRI allows isolation of pathological changes in functional connectivity from other neural effects associated with abnormal phonation, this task-independent approach may have its own technical limitations as a biomarker of SD, because (1) it cannot identify the precise neural locus (rather than inter-regional connectivity) responsible for malfunctioning vocalization and (2) resting-state connectivity measures only have a weak diagnostic power because of their large between-subject variability^15,16^. Accordingly, the existing neuroimaging data have not yet fully clarified the core neural correlates of SD. As described above, the large between-subjects variability in both vocalizations and fMRI signals is likely to confound the statistical comparisons between SD and controls, which may be responsible for the seemingly inconsistent results across previous studies.

In the present study, we used event-related fMRI to examine neural activation during speech perception in patients with “adductor-type” SD, i.e., the most homogenous and prevalent form of SD that represents more than 85% of the entire disease population^17,18^. We employed a sound discrimination task in which SD patients and healthy controls made auditory judgments about normal voice, falsetto and noise (see “Methods” section). It is important to note that these SD patients are particularly impaired in vowel sound production but are relatively unimpaired in falsetto production^5^, probably because falsetto phonation is mainly controlled by the cricothyroid muscle and only weakly relies on glottal adduction in comparison with normal voice^19^. We chose the auditory perceptual task because some recent studies of focal dystonia point to abnormal sensory-motor integration as a generator mechanism of muscular hyperactivity^20–22^. According to this “sensorimotor integration model” of focal dystonia, it is possible that SD patients exhibit abnormal sensorimotor coupling not only during speech production but also during speech perception. This hypothesis likely resonates with the well-known “motor theory of speech perception,” whereby the motor system for vocalization is automatically activated during speech perception^23^. In fact, speech perception plays an important role in sensory feedback to vocalization^24,25^.

At the neural level, the production and perception of speech sounds are also known to activate largely overlapping brain regions, including the left frontotemporal cortex with the inferior frontal, prefrontal, and superior temporal areas^26–30^. Thus, it should be expected that mere exposure to speech sounds can engage these brain regions involved in speech production, which may show different levels of activation in SD patients and healthy participants. According to the sensor-motor model of focal dystonia, we predicted that brain activation patterns during speech perception should differ between SD patients and healthy participants for modal voices but not for falsetto voices. The present experimental design required no overt spoken response and thus allowed us to isolate the neuroanatomical locus of sensorimotor integration issues in SD, while eliminating any neural effects associated with spoken production. Furthermore, we investigated whether fMRI signals during speech perception can serve as a specific biomarker reflecting the pathophysiology of SD. First, we examined whether clinical disease severity measures are correlated with neural activation levels in the sensorimotor and subcortical regions previously associated with SD. Second, we assessed the diagnostic potential of the same fMRI data in distinguishing SD from healthy controls by using machine learning methods.

## Results

### Behavioral data

Mean error rates (standard deviation) during sound discrimination were 0.5% (1.0%) for voice, 1.0% (1.7%) for falsetto, and 6.8% (5.4%) for noise in the SD group and 0.5% (1.0%) for voice, 0.3% (0.8%) for falsetto, and 5.3% (6.4%) for noise in the control group.

### Brain imaging data

**Figure 1:**
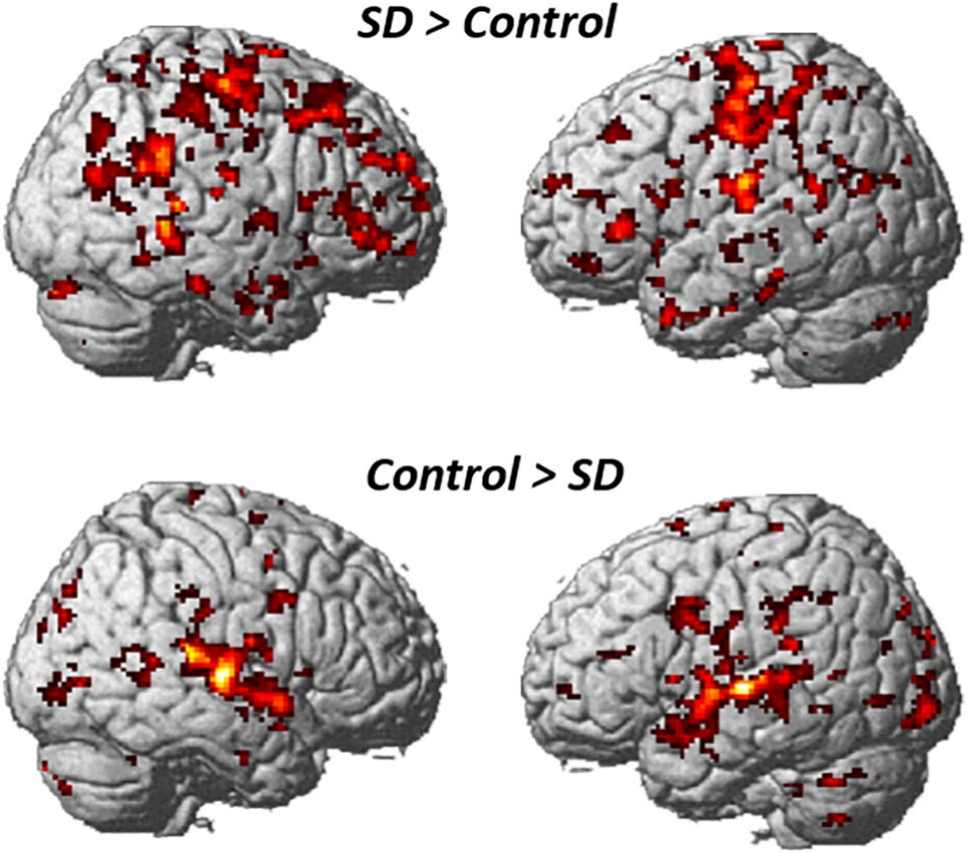
Brain regions showing the main effects of group (SD vs. control). The main effect of group (SD > control) was observed in the left sensorimotor area extending to the SMA, whereas the opposite contrast revealed left superior temporal gyrus activation.

The sound discrimination task strongly activated the superior temporal gyrus and the transverse temporal gyrus in both hemispheres relative to the baseline, consistent with previous studies showing bilateral superior temporal gyrus activation during voice perception as compared with non-voice perception in healthy humans^30–32^. The main effect of group (SD > control) was observed in the left sensorimotor cortex extending to the SMA (− 42, − 22, 64, Z > 8, − 66, − 12, 22, Z > 8), whereas the opposite contrast revealed left superior temporal gyrus activation (− 40, − 28, 6, Z > 8, − 52, − 26, 8, Z = 7.68) (Fig. 1).

**Figure 2:**
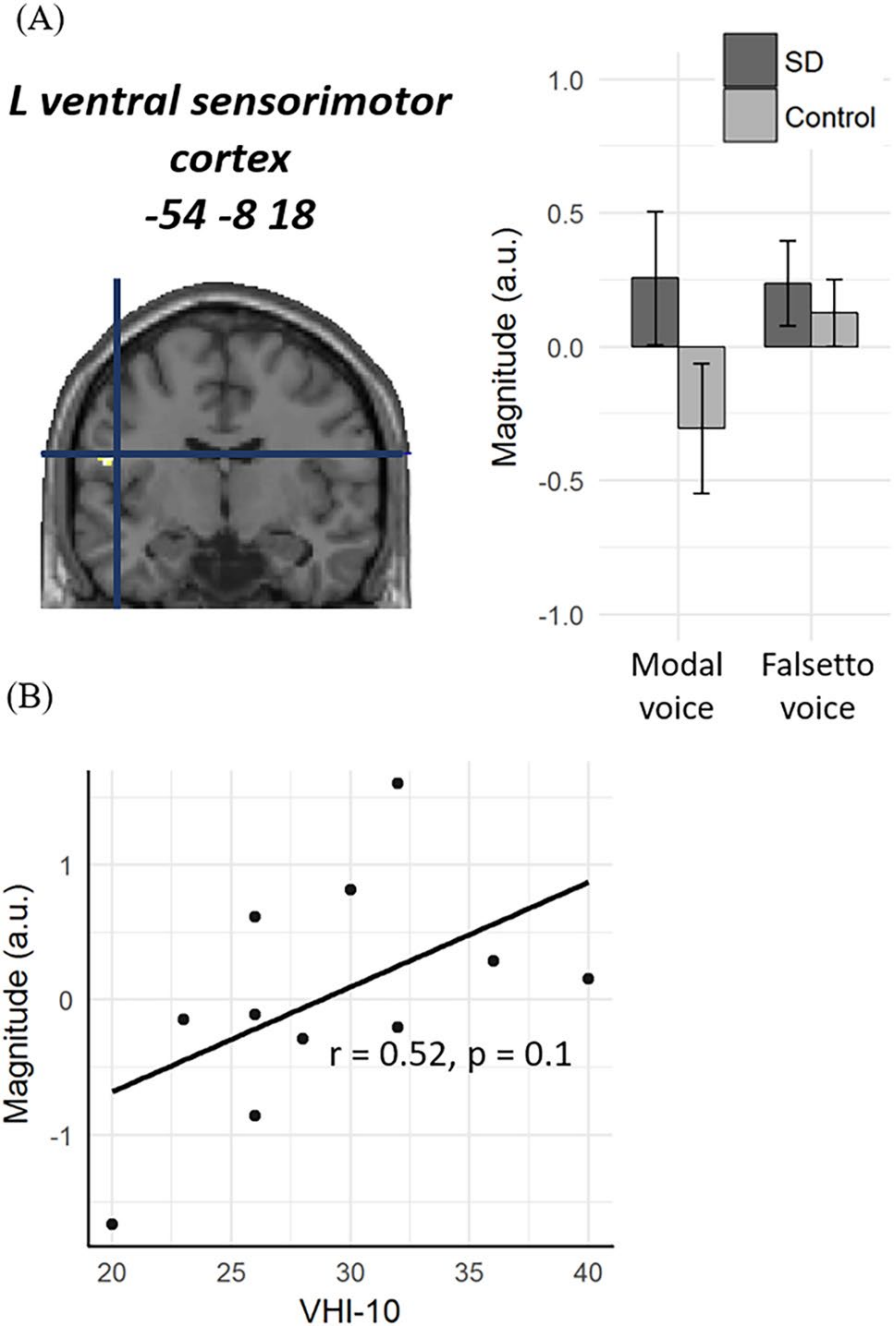
Stimulus-by-group interaction in the ventral sensorimotor cortex. (**A**) Only the left ventral sensorimotor cortex showed significant interaction between stimulus and groups in whole-brain SPM. Note that this region showed greater activation to modal voice relative to falsetto voice, whereas this effect of stimulus was greater for SD than for controls. (**B**) fMRI signals in the same ventral sensorimotor cortex showed no significant correlation with symptom severity.

To identify specific neuroanatomical correlates of SD, we then examined the interaction between the effects of stimulus type (modal voice vs. falsetto voice) and group (SD vs. control). This stimulus × group interaction was significant only in the left ventral sensorimotor cortex (− 54, − 8, 18, Z = 3.90, Fig. 2A), which showed a greater effect of group (SD > control) for modal voice than for falsetto voice. When the analysis was restricted to the modal voice condition, this left sensorimotor region showed greater activation for SD patients than for controls (Z = 3.82). In contrast, this between-group difference was non-significant for the falsetto voice condition (Z < 1.70). Therefore, these findings indicate that (1) this part of the left sensorimotor cortex was more active in SD than in control and that (2) this effect of group was significant only for modal voice and not for falsetto voice.

**Figure 3:**
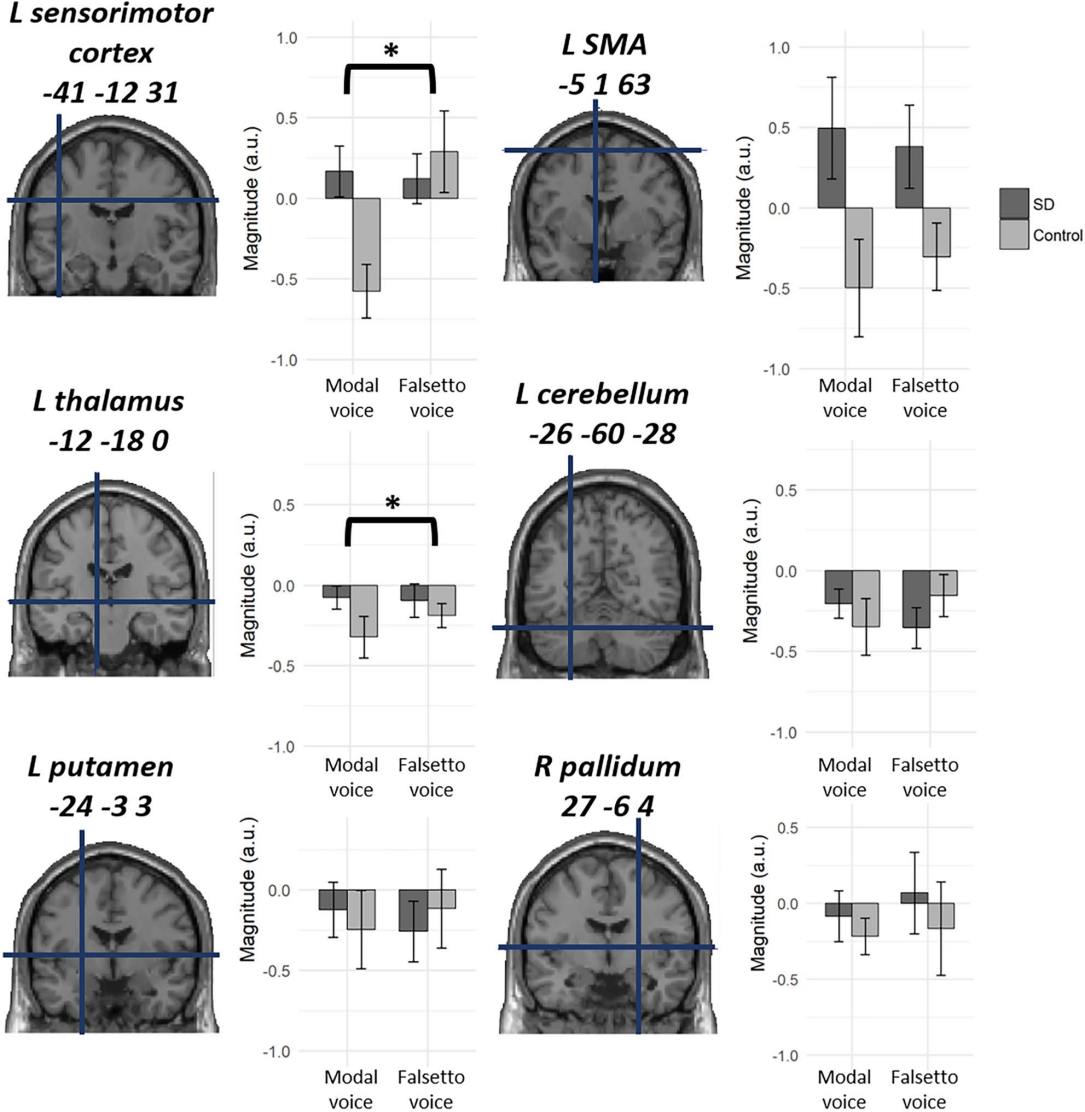
Stimulus-by-group interaction in priori ROIs associated with SD. The left sensorimotor cortex showed significant interaction between stimulus and group. The same effect was also significant in the left thalamus. The SMA showed greater activation in SD relative to controls irrespective of stimulus type and thus showed no significant interaction (see “Results” section for detail).

We then looked at activation patterns in four other regions previously associated with SD, i.e., the left sensorimotor cortex (− 41, − 12, 31), SMA (− 5, 1, 63), thalamus (− 12, − 18, 0), cerebellum (− 26, − 60, − 28), putamen (− 24, − 3, 3), and the right pallidum (27, − 6, 4) (Fig. 3). Although these cortical and subcortical structures did not survive the whole-brain SPM analysis as described above, we ran this ROI analysis to assess the stimulus x group interaction more closely for these regions by using a priori known coordinates of ROIs (see “Methods” section). As for the magnitude of neural activation, the ROI analysis revealed a significant stimulus x group interaction in the left sensorimotor cortex (Z = 3.36, *p* = 0.03) and in the left thalamus (Z = 3.09, *p* = 0.02). The same interaction was not significant in the cerebellum (Z = 2.54, *p* = 0.08), SMA (Z = 1.92, *p* = 0.2), putamen (Z = 1.65, *p* = 0.3) and pallidum (Z = 1, *p* = 0.5). Consistent with the whole-brain SPM, the left SMA showed greater activation to both modal voice and falsetto voice for SD patients relative to controls. This pattern of neural response is thus non-specific to the nature of speech stimuli and is unlikely to reflect the primary pathophysiological focus of the disease, because, as described above, SD patients show impaired vowel sound production but relatively unimpaired falsetto production^5^. Rather, this finding may be attributed to some secondary neural changes of the disease^33^, since the SMA is known to play a role in motor planning during speech production.

Given the observed stimulus × group interaction in the sensorimotor cortex and the thalamus, we additionally examined possible changes in functional connectivity in SD patients. Indeed, this PPI analysis revealed that the functional coupling between the thalamus (− 12, − 18, 0) and the left sensorimotor cortex (− 41, − 12, 31) showed a significant decrease in SD compared with controls (Z = 3.34, *p* = 0.01). Coupled with whole-brain SPM analysis, these findings thus suggest that the left sensorimotor area corresponds to the “human voice area”^34^ and the thalamus plays a specific role in SD pathophysiology.

**Figure 4:**
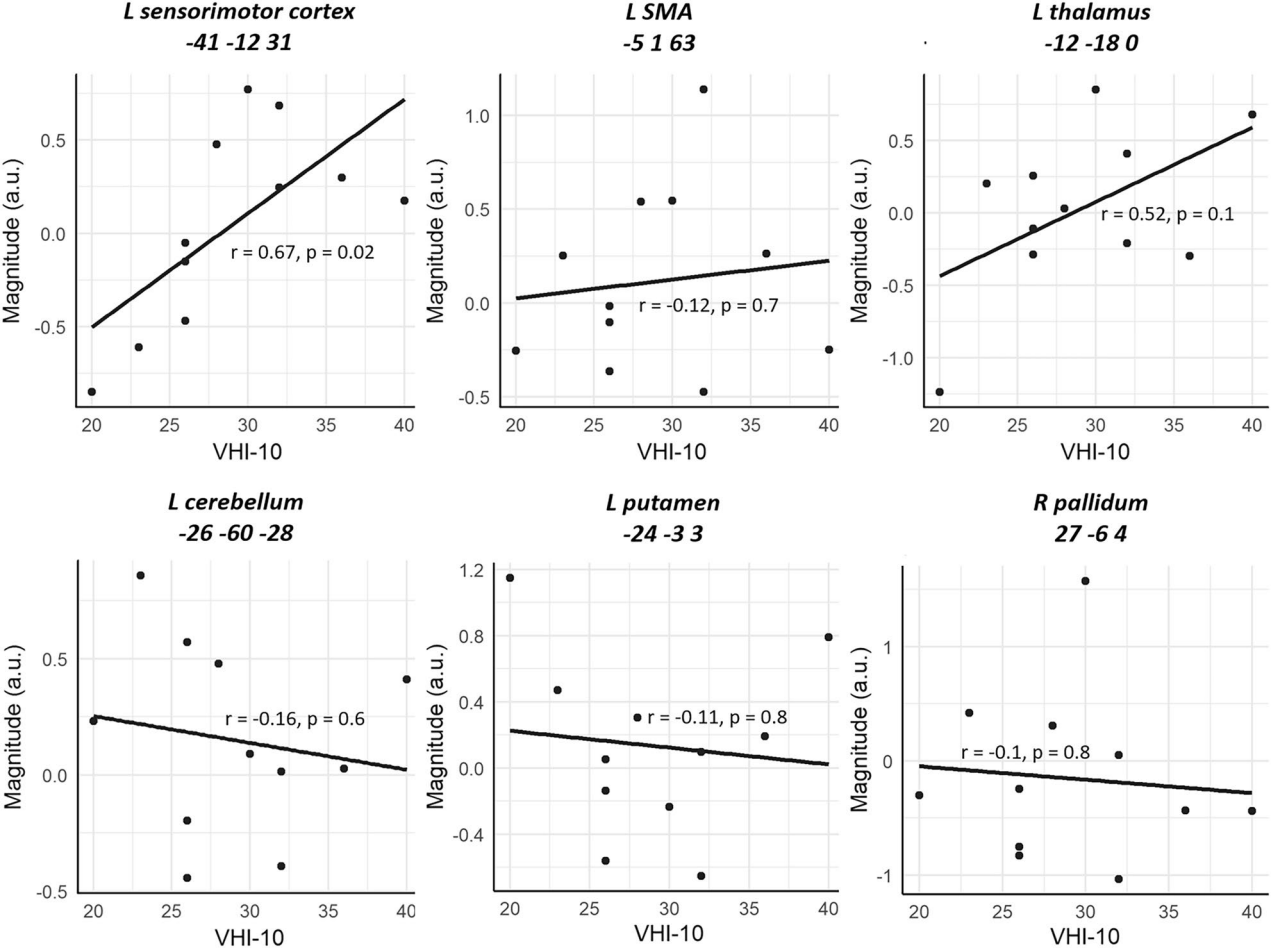
Correlations between fMRI signals and symptom severity. For each region, the magnitude of activation (modal voice and falsetto voice) is plotted against disease severity. The magnitude of activation (modal voice > falsetto voice) showed a positive correlation with VHI-10 scores only in the left sensorimotor cortex.

For SD patients, we further looked at possible correlations between fMRI signals and disease severity by plotting the activation level of the left ventral sensorimotor cortex (− 54, − 8, 18) identified in the SPM analysis against individual VHI-10 scales (Fig. 2B). We observed non-significant correlation between fMRI signals and the VHI-10 scores (r = 0.52, *p* = 0.1). In Fig. 4, we further plotted the magnitude of activation against the individual VHI-10 score for each of the six spherical ROIs created at a priori known coordinates. This supplemental analysis revealed significant positive correlations between neural signals in the left sensorimotor cortex and VHI-10 scores (r = 0.67, *p* = 0.02). For other regions, however, the correlations between neural signals and disease severity were non-significant (r = − 0.12, *p* = 0.7 for the SMA; r = 0.52, *p* = 0.1 for the thalamus; r = − 0.16, *p* = 0.6 for the cerebellum; r = − 0.11, *p* = 0.8 for the putamen; and r = − 0.1, *p* = 0.8 for the pallidum). While several cortical and subcortical regions have been associated with SD, these findings therefore suggest that only the neural signals from the sensorimotor cortex specifically reflect the clinical severity of the disease.

**Figure 5:**
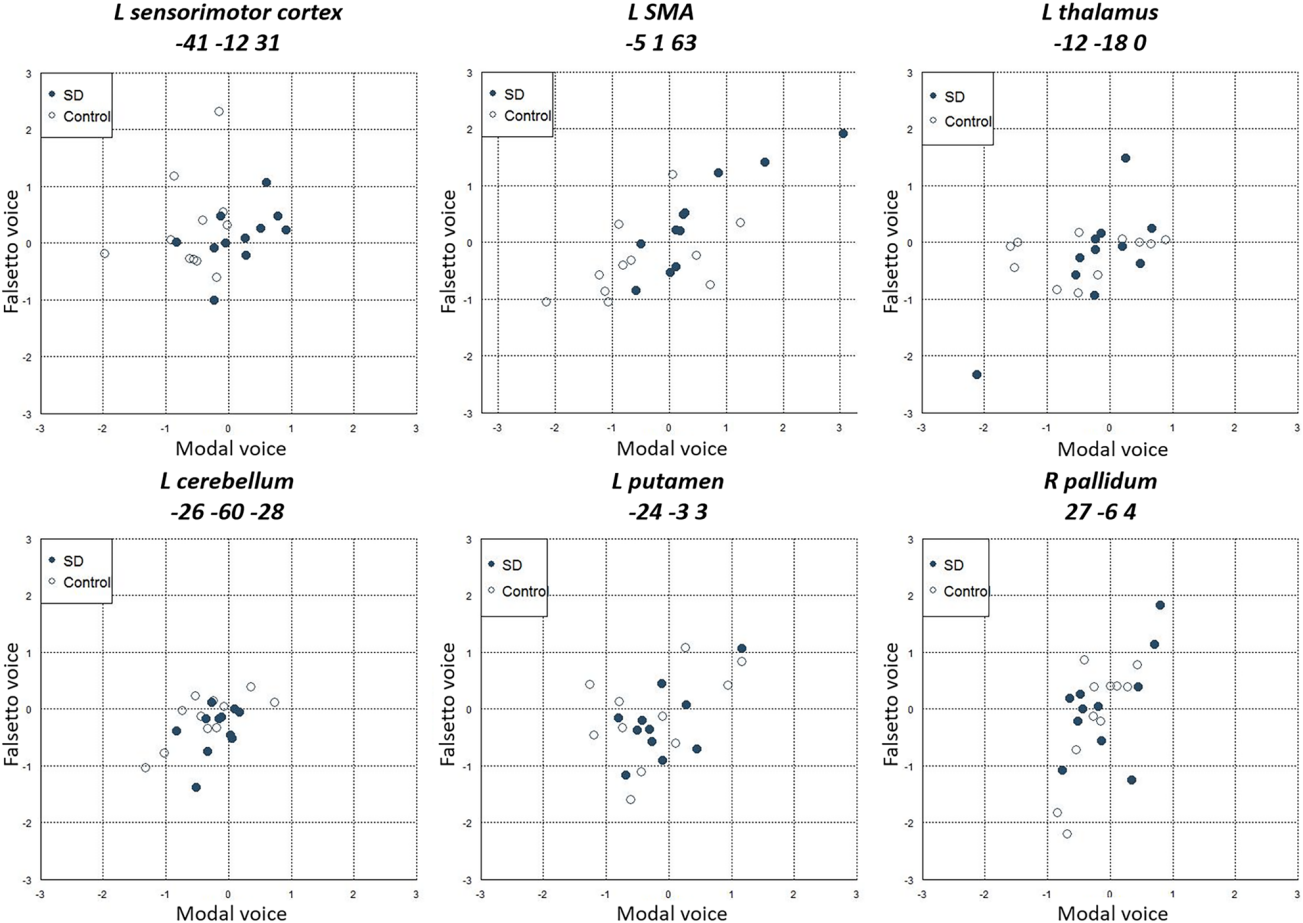
Scatterplots of fMRI signals in four ROIs. For each region, the magnitude of activation for modal voice (relative to noise) is plotted against that for falsetto voice (relative to noise).

Lastly, we assessed the diagnostic power of fMRI to separate SD patients from healthy controls. For each ROI, individual activation signals were used to train and test three different machine learning algorithms (see “Methods” section and Fig. 5). The classification performance measures for each method are summarized in Table 1. Classification accuracy was consistently higher for the sensorimotor cortex (> 59%) compared to other three regions (~ 59% or chance-level). In particular, linear discriminant analysis for this region achieved 73% accuracy and yielded the highest performance in terms of precision, specificity, recall, and F-measures. These performance metrics seem to exceed those reported in a previous fMRI study that classified SD patients and healthy controls with 71% accuracy by using functional connectivity measures^14^. The present findings suggest that sensorimotor activation signals can help to distinguish SD patients from healthy controls accordingly.

**Table 1:** Machine learning to evaluate the diagnostic significance of ROI activity.

	Accuracy (%)	Precision (%)	Recall (%)	Specificity (%)	F-measure (%)
**Linear discriminant analysis**					
Sensorimotor	73	69	82	64	75
SMA	59	58	64	55	61
Thalamus	27	27	27	27	27
Cerebellum	41	42	45	36	43
Putamen	32	30	27	36	29
Pallidum	23	25	27	18	26
**Support vector machine**					
Sensorimotor	59	60	55	64	57
SMA	50	50	55	45	52
Thalamus	59	60	55	64	57
Cerebellum	50	50	55	45	52
Putamen	30	31	36	25	33
Pallidum	32	33	36	27	35
**Logistic regression**					
Sensorimotor	68	67	73	64	70
SMA	57	54	64	50	58
Thalamus	32	33	36	27	35
Cerebellum	43	42	45	42	43
Putamen	32	30	27	36	29
Pallidum	23	25	27	18	26

## Discussion

The present study used fMRI to isolate specific neural correlates of SD during speech perception. While most previous brain imaging studies of SD employed overt vocalization tasks, a critical problem inherent in such production tasks is that normal and dystonic vocalizations each should differently engage the speech control system at multiple neural levels (i.e., cognitive, motor, somatosensory, and auditory), which could confound statistical comparisons between the SD and control groups. This may explain the fact that previous neuroimaging studies have reported variously different patterns of sensorimotor activation in SD^9–11^. In the present study, we used a sound discrimination task to overcome the potential drawback associated with speech production and obtained similar level of behavior performance between SD patients and healthy controls.

In SPM analysis, however, we observed different patterns of neural activation in the left ventral sensorimotor cortex between the two groups, i.e., the nature of speech stimuli (modal voice vs. falsetto voice) elicited a much weaker impact on SD patients than on healthy controls (Fig. 2A). This same stimulus-by-group interaction was also obtained in the left sensorimotor ROI previously associated with laryngeal adduction in SD (Fig. 3). These results are in good accord with a well-known and time-honored clinical feature of adductor-type SD, i.e., its “task-specificity,” where falsetto phonation is free of voice breakage^5^, and may concur with some neuroimaging studies of SD and other dystonic disorders^9,35,36^. These findings seem consistent with previous TMS studies showing that the hyperactive somatosensory cortex increases the neuromuscular excitability of the speech production system in SD^12,13^. Together, the present findings converge to suggest that reduced sensorimotor reactivity is a primary neural source responsible for the voice disorder in SD.

Furthermore, we found that the activation level of the sensorimotor area positively correlated with symptom severity as measured with VHI-10 scores, providing additional support for the notion that sensorimotor overactivity is a key component underlying voice disorder in SD. This may also explain the finding that sensorimotor signals discriminated SD patients from controls with high accuracy (73%), which exceeded the classification performance reported by Battistella^14^. These findings suggest that the fMRI signal of the left sensorimotor cortex can serve as a good index of disease severity and a diagnostic clue for SD.

On the other hand, our ROI analysis revealed that the left thalamus exhibited a significant but different pattern of stimulus-by-group interaction, with the effect of stimulus being weaker from the baseline (Fig. 3). The observed difference in activation profile suggests that the sensorimotor cortex and the thalamus each play a different role in the pathophysiology of SD. This seems to be consistent with the proposal that subcortical structures, including the basal ganglia and thalamus, play a key part in abnormal sensorimotor integration in movement disorders^20^. Using PPI analysis, we indeed observed decreased functional connectivity between the thalamus and the sensorimotor cortex. This latter finding seems to be consistent with previous diffusion-tensor MRI studies showing impaired functional connectivity between the thalamus and precentral gyrus in idiopathic dystonia^37,38^. This disrupted thalamo-cortical connectivity is thought to reflect intrinsic abnormal neuronal firing within the thalamus and the precentral gyrus^37^. Coupled with the present finding, reduced thalamo-sensorimotor coupling may play a role in the generation of abnormal vocalization in SD.

However, unlike the sensorimotor cortex, the left thalamus ROI showed no significant correlation between fMRI signals and disease severity. A plausible account for this finding is provided by recent neuroimaging and neuronal recording studies suggesting that ventrolateral thalamic activity reflects the strength of sensory afferents from the proprioceptive receptors in muscles, tendons, and joints^39,40^, rather than the neuromuscular excitability of laryngeal muscles. That is, thalamic activations are primarily driven by peripheral inputs from deep sensory cells and thus only weakly correlated with increased muscle activity in SD. Compared to subcortical signals, sensorimotor activation levels may be directly correlated with symptom severity, since phonation itself strongly depends on fine orchestration of multiple cortical networks^14^.

In conclusion, the present study identified the sensorimotor cortex and the thalamus as the primary neural correlates of SD by using the sound discrimination task without overt vocalization. Specifically, the neural activation level of the sensorimotor cortex was elevated in SD relative to controls and correlated with disease severity. Our findings suggest that fMRI signals from these structures may serve as a novel biomarker for SD, but leave at least two major questions to be addressed in future research. First, it is still open whether fMRI signals have the potential to differentiate SD from other functional voice disorders, such as psychogenic voice disorder and muscle tension dysphonia, which mimic the strained and effortful voice characteristics of adductor-type SD and thus lead to diagnostic confusion and treatment delay^3,4,6^. It is therefore an important clinical problem to improve the diagnostic precision for SD, because therapeutic strategies are radically different between adductor-type SD and other disorders (i.e., surgery or botulinum toxin injections for SD and psychologic or voice therapy for other disorders). While several perceptual scales, such as VHI-10, GRBAS scale, CAPE-V, and number of voice break, are now available for assessing symptom severity^41–43^, “gold standard” criteria for differential diagnosis has yet to be established. In future research, it is thus important to determine the extent of the diagnostic power of fMRI signals in SD and other voice disorders. Second, it also remains unclear whether the observed hyperactivity in the sensorimotor cortex plays a causal role in the pathophysiology of SD or rather it reflects some plastic change in the brain after abnormal vocalization. As for other forms of dystonia, previous TMS studies show a causal link between the somatosensory cortex and writer’s cramp^44,45^. Arguably, abnormal sensorimotor activation may be also causally linked with SD, but the causal relationship between SD and motor cortical excitability has not yet been established because the existing TMS data show a shorter cortical silent period in patients than healthy controls, which only suggests some impairment in inhibitory control in the corticobulbar and cortical spinal tracts^12,13,46^. Further studies are needed to investigate whether TMS stimulation to the left sensorimotor cortex improves the dystonic voice and clarify the potential and limits of fMRI signals in the clinical diagnosis of SD.

## Methods

### Participants

Eleven adductor-type SD patients (two males; age range, 21–68 years; average, 36.7 years) and 11 age- and gender-matched healthy participants (two males; age range, 21–65 years; average, 30.9 years) participated in the present study (Table 2). All of them were right-handed native Japanese speakers with normal hearing and normal or corrected-to-normal vision. None of the patients had a known family history of dystonia and neurological or psychiatric disorders other than SD. The mean age at onset and duration of SD was 26.1 years (range, 9–58 years) and 8.5 years (range, 3–20 years), respectively. No patients had received botulinum toxin injections into their laryngeal muscles for the control of voice breakage. The clinical diagnosis of SD was made by certified otolaryngologists (Otorhinolaryngological Society of Japan). The patients fulfilled all the following criteria: (1) a strained/strangled voice with intermittent disruption^47^, (2) hyperadduction of the vocal folds during voice breakage, (3) no anatomic abnormality of the larynx observed on fiberoptic laryngoscopy, and (4) poor improvement in spite of voice therapy. All patients reported that they could produce falsetto voice without difficulty. We further confirmed that all patients could vocalize falsetto /u:/ without voice breakage. Clinical evaluation of severity was performed using the voice handicap index-10 (VHI-10)^48,49^. In brief, the VHI-10 is a patient-based self-assessment tool widely used to quantify the severity of voice problems in physical, mental and social aspects for a variety of voice disorders^50,51^. The mean VHI-10 score was 28.7 (range, 20–40) for SD patients and 4.9 (range, 0–13) for healthy controls, respectively. We also used the VHI-10 scores to evaluate their correlation with fMRI signals (see “Statistical analyses” section). While symptom severity of voice disorders can also be assessed with auditory-perceptual testing (e.g., voice break counts, GRBAS scale, CAPE-V), VHI-10 scores were used because it considers symptom variability. We assumed that this patient-reported outcome measure is a more stable estimate of overall severity than other physiological or behavioral measurements at a single test point. This is important given the fact that clinical symptoms in SD can easily fluctuate with test settings^5,52,53^. All participants provided written informed consent prior to the experiment. The protocol of this study was approved by the ethics committee of Kyoto University Hospital (C 1041). The study protocol was conducted in accordance with the Declaration of Helsinki.

**Table 2:** Participant characteristics.

	SD patients	Healthy controls
Number of participants	11	11
Gender (women/men)	9/2	9/2
Average age (range)	36.7 (21–68)	30.9 (21–65)
Age of onset (range)	22 (9–58)	n/a
Duration of illness (year, range)	7 (3–20)	n/a
Average VHI-10 score (range)	28 (20–40)	4.9 (0–13)

*VHI-10* voice handicap index-10, *n/a* not applicable.

### Behavioral tasks

We used a two-alternative sound discrimination task to measure event-related fMRI activations in the neural systems involved in speech processing. We recorded two monosyllabic sounds, /a:/ and /i:/, each pronounced in two different voices (modal and falsetto) by a female native Japanese speaker (see “Supplementary Materials”). An additional pair of physical control stimuli was created by using “white noise” containing all frequencies from 20 to 11,000 Hz and “band noise” with a center frequency of 1000 Hz. All of the six sound stimuli were sampled at 44 kHz, and their overall sound pressure was adjusted to be equal to each other. Auditory stimuli were presented with the E-prime 2.0 software (Psychology Software Tools, Pittsburgh 2000). Each trial started with a variable jitter interval (3–7 s, in steps of 1 s) and included a sound stimulus (modal voice, falsetto voice, or noise) presented for 2 s. The three types of trials (modal voice, falsetto voice, and noise) were presented to participants in a pseudo-random order. Participants responded with their right index finger by pressing either (1) a right button for the vowel /a:/ (modal voice or falsetto voice) or the white noise or (2) a left button for the vowel /i:/ (modal voice or falsetto voice) or the band noise. Each participant received two scanning sessions, each consisting of 60 trials (thus 20 trials for each modal voice, falsetto voice, and noise conditions) and lasting 432 s. Before MRI scanning (see below), each participant performed practice trials with the same trial structure outside and inside the scanner.

### Imaging procedure

Imaging data were acquired using a Siemens Trio 3 T head scanner with a 32-channel phased-array head coil. The echo planer imaging (EPI) data were acquired using a recently developed multiband EPI sequence^54^: TR = 1.0 s, TE = 30 ms, flip angle = 90°, field of view (FOV) = 192 mm × 192 mm, multiband acceleration factor = 4, voxel size = 3 × 3 × 3 mm, and 60 axial slices. The 3-dimensional T1-weighted images with MPRAGE sequence were acquired with following parameters: TR = 2.0 s, TE = 3.37 ms, inversion time = 990 ms, FOV = 256 mm, voxel size = 1 × 1 × 1 mm, flip angle = 8°, and 130-Hz bandwidth. Participants underwent two scanning sessions, each lasting 432 s.

### Statistical analyses

Imaging data were preprocessed and analyzed using the Statistical Parametric Mapping (SPM8; Wellcome Department of Cognitive Neurology, London, UK) package software run on Matlab R2014a. Functional images were corrected for head movement, normalized to the standard brain space by the Montreal Neurological Institute (MNI) space using T1 image unified segmentation (the resampling voxel size was 2 × 2 × 2 mm), and smoothed with a 4-mm Gaussian kernel. For the first-level analysis, we assessed the functional images by constructing a general linear model with a factor of three levels: modal voice, falsetto voice, and noise. High-pass temporal filtering (128 Hz) was applied to the fMRI time-series data. For each participant, three contrast images (modal voice, falsetto voice, and noise) were calculated relative to the baseline condition by convolving known time-series of trials with a canonical hemodynamic response function and its time derivative.

In the second-level analysis, we submitted the three contrast images per participant to a 2 × 2 analysis of variance (ANOVA) to examine the effects of stimulus type (modal voice, falsetto voice) and group (SD and control) on brain activation. We first delineated brain areas involved in voice processing by collapsing the contrast images of “modal voice > noise” and “falsetto voice > noise” from all participants across the two groups. We then searched for the effects of stimulus and group and their interactions within this set of voxels involved in voice processing. Unless stated otherwise, statistical significance was examined with the voxel-level threshold *p* < 0.001 uncorrected (extent threshold = 10 voxels). Activated brain regions were identified according to a probabilistic atlas^55^. Talairach coordinates referred from previous studies were converted into MNI spaces using the Yale Nonlinear Talairach to MNI Conversion Algorithm^56^.

To examine the critical stimulus (modal voice vs. falsetto voice) × group (SD vs. control) interaction more closely, we performed region of interest (ROI) analyses for four cortical and subcortical regions previously associated with SD, i.e., the left sensorimotor cortex^9–11,33^, the left SMA^10,11,33,57^, thalamus^9,33^, lobule IV of the cerebellum^9–11,33,57,58^, putamen^9^ and pallidum^9^. We created a spherical ROI with a 4-mm radius at each of the known coordinates of the left sensorimotor cortex (− 41, − 12, 31)^34^, the left SMA (− 5, 1, 63)^34^, the left thalamus (− 12, − 18, 0)^59^, and lobule VI of the left cerebellum (− 28, − 60, − 26)^33,34^, the left putamen (− 24, − 3, 3)^59^, the right pallidum (27, − 6, 4)^59^. For each ROI, we examined the stimulus-by-group interaction with a voxel-level threshold *p* < 0.05 corrected for multiple comparisons.

We performed psychophysiological interaction (PPI) analysis to explore the possible changes in cortico-subcortical connectivity in SD, because previous studies have demonstrated that this connectivity is impaired in dystonic diseases including SD^14^. In brief, PPI has the potential to evaluate neural coupling of one area to another affected by an experimental or psychological context^60^. Among the cortical areas, we chose the left sensorimotor cortex (− 41, − 12, 31), which was identified as an SD-specific region from the group analysis, and extracted regional responses per participant by calculating the principal eigenvariate across all voxels within a 4-mm sphere centered at the sensorimotor area. We then performed regression analysis for time-series data of the sensorimotor activity by computing the PPI regressor and a vector coding for the differential effect across stimulus types (1 for modal voice, − 1 for falsetto voice) per session per participant. The contrast image for stimulus type (i.e., “modal voice > falsetto voice”) created per participant was submitted to second-level analysis (two-sample *t* test) to examine the group effect (SD vs. control). We created a spherical ROI with a 4-mm radius at the known coordinates of the left thalamus (− 12, − 18, 0) identified as the SD-specific cortical region from group analysis and examined the group effect at the voxel level with the threshold *p* < 0.05 corrected for multiple comparisons across search volume.

We further performed two supplemental analyses to assess whether these fMRI signals during speech perception can serve as a clinically informative biomarker for SD. First, we examined whether clinical disease severity measures are correlated with neural activation levels in these cortical and subcortical regions associated with SD. Using fMRI signals extracted from the spherical ROIs described above, we calculated Pearson’s correlation strength to examine whether the effect-size of stimulus type (modal voice > falsetto voice) correlated with disease severity as measured with VHI-10. Second, we used machine learning algorithms to examine whether the fMRI data can distinguish SD patients from healthy controls. Three linear classification methods, i.e., linear discriminant analysis, linear support vector machine, and logistic regression, were performed using the Caret package in R (https://www.r-project.org/). In each classification analysis, we extracted individual-level activation signals derived from modal voice versus noise and falsetto voice versus noise comparisons by using the same spherical ROIs described above. We then performed a 26-fold leave-one-out cross-validation procedure that used all individual fMRI data except one to train the classifier and the remaining data to evaluate the prediction accuracy, respectively. Other metrics, i.e., precision, recall, specificity, and F-measure^61^ were also calculated to compare classification performance across the three models.

## Supplementary information

Supplementary Information 1.

Supplementary Information 2.

Supplementary Information 3.

Supplementary Information 4.

## Data Availability

The data that support the findings of this study are available from the corresponding author, upon reasonable request.
